# Use of lacosamide in children: experience of a tertiary medical care center in Brazil

**DOI:** 10.1055/s-0042-1758366

**Published:** 2022-12-28

**Authors:** Tayla Taynan Romão, Abraão Iuri Medeiros Angelim, Henrique Jannuzzelli Pires do Prado, Fernanda Veiga de Goes, Maria Elisa Paiva Pires, Rosiane da Silva Fontana, Lais de Carvalho Pires, Alexandre Ribeiro Fernandes, Isabella D'Andrea-Meira

**Affiliations:** 1 Universidade Federal Fluminense, Faculdade de Medicina, Departamento de Neurologia, Niterói RJ, Brazil.; 2 Instituto Estadual do Cérebro Paulo Niemeyer, Departamento de Epilepsia, Rio de Janeiro RJ, Brazil.; 3 Instituto Nacional de Saúde da Mulher, da Criança e do Adolescente Fernandes Figueira, Departamento de Neurologia, Rio de Janeiro RJ, Brazil.

**Keywords:** Epilepsy, Lacosamide, Child, Drug Resistant Epilepsy, Pediatric Assistants, Epilepsia, Lacosamida, Criança, Epilepsia Resistente a Medicamentos, Assistentes de Pediatria

## Abstract

**Background** Lacosamide (LCM) is a third-generation anti-seizure drug approved in Europe and the United States, either as a monotherapy or adjunctive therapy, to treat partial-onset seizures in adults, adolescents, and children. In Brazil, LCM is licensed for treatment only in patients older than 16 years of age.

**Objective** To evaluate a cohort of children presenting with refractory epilepsy who received LCM as an add-on therapy and observe the response and tolerability to the LCM treatment.

**Methods** A retrospective cohort study conducted in a tertiary health care facility, which included 26 children, aged up to 16 years, who presented with refractory epilepsy and received LCM as an add-on treatment. The follow-up visits were scheduled every 3 months until 9 months of treatment with LCM.

**Results** After 3 months of LCM administration, in 73.1% of the children, there was a reduction of > 50% in the frequency of seizures, and this clinical improvement was maintained in most patients (73.9%) for the following 9 months. Mild (such as, somnolence and behavioral changes) or severe (seizure worsening) adverse effects were observed in two and three children respectively. Among responders to LCM, there was a higher prevalence of males, fewer concomitant anti-seizure drugs, and lower percentage of patients using sodium channel blockers.

**Conclusions** Lacosamide should be considered as an early treatment option in pediatric patients with refractory epilepsy, mainly focal seizures.

## Resumo

**Antecedentes** Lacosamida (LCM) é um fármaco anticrise de terceira geração aprovado na Europa e nos Estados Unidos, utilizado como monoterapia ou terapia adjuvante para tratar crises epilépticas focais em adultos, adolescentes e crianças. No Brasil, a LCM só é aprovada para tratamento em pacientes com mais de 16 anos de idade.

**Objetivo** Avaliar uma coorte de crianças com epilepsia refratária que receberam LCM como terapia adjuvante e observar a resposta e tolerabilidade ao tratamento.

**Métodos** Um estudo de coorte retrospectivo conduzido em uma unidade terciária de saúde, que incluiu 26 crianças de até 16 anos de idade que apresentavam epilepsia refratária e receberam um tratamento complementar com LCM. As visitas de acompanhamento foram agendadas a cada 3 meses, até 9 meses de tratamento com LCM.

**Resultados** Após 3 meses de administração de LCM, em 73,1% das crianças, a frequência das crises teve uma redução maior do que 50%, e essa melhora clínica foi mantida na maioria dos pacientes (73,9%) pelos 9 meses seguintes. Efeitos adversos leves (como, sonolência e alterações comportamentais) ou graves (agravamento das crises) foram observados em duas e três crianças, respectivamente. Entre as crianças que responderam ao tratamento com LCM, houve uma maior prevalência do sexo masculino, o uso de um menor número de medicações anticrise associadas e o uso de bloqueadores dos canais de sódio.

**Conclusões** A LCM deve ser considerada uma opção de tratamento precoce em pacientes pediátricos com epilepsia refratária, principalmente aqueles que apresentam crises focais.

## INTRODUCTION

In the last few decades, several anti-seizure drugs (ASDs) have been introduced for the treatment of epilepsy. However, due to the heterogeneous characteristics of epilepsy, choosing the appropriate therapy for each patient is challenging. The prescribed treatment should consider not only the type of seizure, but also the individual characteristics of the patients. Several new ASDs have been introduced with different mechanisms of action, new synergistic combinations, fewer adverse events, and fewer pharmacokinetic drug interactions. 1 These drugs can be used as a monotherapy or an add-on therapy in patients with refractory epilepsy. This group of patients is at a higher risk of psychosocial dysfunctions, injuries, premature death, and sudden unexpected death. Refractory epilepsy also affects the patients' quality of life. According to The International League Against Epilepsy (ILAE), drug resistance is defined as the failure of at least two properly-indicated ASDs to treat epilepsy. 1 2 3

Lacosamide (LCM) is a third-generation ASD 4 that has been approved in Europe and the United States, either as a monotherapy or an adjunctive therapy, for the management of focal-onset seizures, with or without secondary generalization, in adults, adolescents, and children. 5 Several studies 6 7 8 have reported infants and young children diagnosed with focal-onset seizures who have been treated with LCM, which can function in the following two ways: it acts as a selective facilitator of the slow inactivation of voltage-gated sodium channels (VGSCs) and possibly binds to collapsin response mediator protein-2 (CRMP-2). 4 9 Lacosamide acts by enhancing slow VGSC inactivation to initiate the generation and propagation of nerve action potentials and neuronal excitability. After depolarization, the VGSCs become inactivated, and further depolarization cannot occur until the VGSCs return to their resting potentials. 5 In patients with epilepsy, sustained depolarization occurs with prolonged high-frequency trains of repetitive firing. In this scenario, the pore of the sodium channel undergoes a structural rearrangement, which results in a slow inactivated state. Lacosamide enhances this transition of VGSCs into a slow-inactivated state and consequently reduces repetitive neuronal firing without affecting the fast inactivation. 5 9 Therefore, its mode of action is different from that of the traditional sodium channel-blocking ASDs.

Lacosamide is available in oral and intravenous (IV) formulations. 10 It is metabolized by the cytochrome P450 (CYP)2C19, 2C9, and 3A4 enzymes into the pharmacologically inactive O-desmethyl lacosamide, with minimal drug interactions and good safety profile. 5 Approximately 95% of the LCM dose is eliminated in the urine. The most common effects related to LCM administration are dizziness, nausea, vomiting, diplopia, vertigo, coordination abnormalities, and blurred vision, which occur more often during the titration period. 6 Interestingly, differences in the physiological processes between neonates and children can influence the distribution of pharmacological drugs. In addition, immature metabolic patterns and differences in renal activity may affect the elimination half-life and, therefore, influence the pharmacokinetics of several ASDs. 2

In Brazil, LCM has been approved for the treatment of focal onset seizures as an add-on or monotherapy in patients older than 16 years of age. 4 Nevertheless, LCM is an off-label drug administered to children. The present study aimed to evaluate a cohort of children presenting with refractory epilepsy who received LCM as an add-on therapy and observe the response and tolerability to the treatment.

## METHODS

### Study design

The present was a retrospective cohort study conducted at a tertiary health care center in Rio de Janeiro, Brazil, the Epilepsy Department of Instituto Estadual do Cérebro Paulo Niemeyer (IECPN). The study protocol was approved by the IECPN Ethics Committee (no. 3.577.369). The requirement to obtain informed consent was waived due to the retrospective design of the study.

We included children younger than 16 years of age who presented with refractory epilepsy to the IECPN and were treated with LCM for a minimum of 3 months, initiated between January 2014 and June 2018. Patients who did not conform to the prescribed dose of LCM, had irregular attendance in the follow-up visits, or had unreliable clinical records were excluded. The present was a single-center study and the off-label use of LCM in children was not a regular practice. All children who met the inclusion criteria within the study period were included. A total of 26 children were assessed.

Children were classified in terms of epilepsy type according to the 2017 International League Against Epilepsy (ILAE) Seizure Classification, 11 which takes into consideration the patient's clinical and complementary exams, such as neuroimaging and electroencephalography (EEG). Based on that, 24 children presented with focal epilepsy and 2, with generalized epilepsy. Among children with focal epilepsy, 17/24 (70.8%) had a structural lesion on the brain magnetic resonance imaging (MRI) scan. The 2 children with generalized epilepsy had normal brain MRI scans.

### Data collection and analysis

Data were collected from the patients' medical records, including: sex, age at the first seizure, duration of epilepsy, etiology and type of epilepsy, type and frequency of seizures, MRI abnormalities, any associated psychiatric condition, the number and type of previous ASDs, and other previous treatments (ketogenic diet, neurosurgery, and vagus nerve stimulation [VNS]). The follow-up visits were scheduled every three months, and data regarding the number of concomitant ASD types, age at the start of LCM administration, initial and final LCM dosages, and adverse effects occurring at 3, 6, and 9 months after LCM initiation were obtained. Data after 3 months of the LCM treatment were only unavailable for 1 patient.

The primary endpoint of the present study was the quantification of the response to the LCM treatment. The parameter used was the frequency of seizures, and its decrease was defined as a reduction of at least 50%. The adverse effects were analyzed with the reported data and divided into two categories: severe adverse effects; and mild/moderate or no adverse effects. Their relationship with the LCM treatment or the concomitant use of other ASDs was also explored. A descriptive analysis was performed for all variables, and mean ± standard deviation (SD) were used for the continuous variables, and absolute and relative frequencies, for categorical variables. Due to the small sample size, non-parametric tests, such as the Mann–Whitney U test for comparisons of means and the Fisher exact test for contingency tables, were used. The univariate analysis and multivariate regression model were used to evaluate the association between characteristics of the children and their response to the treatment. Statistical significance was defined as *p* ≤ 0.05, and all statistical analyses were performed using the Statistical Package for the Social Sciences (IBM SPSS Statistics for Windows, IBM Corp., Armonk, NY, United States) software, version 21.

## RESULTS

### Study population

The sample consisted of 26 children with refractory epilepsy who were treated with LCM as an add-on therapy ( *n* = 24) or monotherapy ( *n* = 2). The first seizure was between 0 and 11 years of age, and most children presented with focal epilepsy (92.3%) and abnormal brain MRI findings (62.3%). The LCM treatment started at a mean of 4.8 ± 3.4 years after the diagnosis of epilepsy ( Table 1 ). The children received at least two and a maximum of nine ASDs prior to the administration of LCM. The most common ASD used was oxcarbazepine, followed by topiramate. Among the participants, 50% (13) of the children had previously undergone adjuvant therapies, varying from 1 to 3 in number, with neurosurgery, ketogenic diet, and immunotherapy being the most common ( Table 2 ).

**Table 1:** Baseline characteristics of the study population

Characteristics		Population ( *n* = 26)
Sex: n (%)	Male	13 (50%)
	Female	13 (50%)
Age at seizure onset (years): mean ± standard deviation		3.9 ± 3.9
Age at the start of the treatment with lacosamide (years): mean ± standard deviation		8.7 ± 3.9
Duration of the epilepsy time at the start of the treatment with lacosamide (years): mean ± standard deviation		4.8 ± 3.4
Abnormal brain magnetic resonance imaging findings: n (%)		17 (65.4%)
Type of epilepsy: n (%)	Focal	24 (92.3%)
	Generalized	2 (7.7%)
Etiology: n (%)	Genetic	2 (7.7%)
	Genetic + structural	7 (26.9%)
	Structural	10 (38.5%)
	Unknown	7(26.9%)
Associated comorbidities: n (%)	Clinical	12 (46.2%)
	Psychiatric	7 (26.9%)

**Table 2:** Characteristics of the previous adjuvant therapies

Characteristics	Population ( *n* = 26)
**Mean number of previous therapies: range (mean ±** **standard deviation)**	2–9 (4.5 ± 2.0)
Oxcarbazepine: n (%)	20 (72.9%)
Topiramate: n (%)	16 (61.5%)
Levetiracetam: n (%)	14 (53.8%)
Valproic acid: n (%)	14 (53.8%)
Phenobarbital: n (%)	10 (38.5%)
Lamotrigine: n (%)	9 (34.6%)
Clobazam: n (%)	7 (26.9%)
Clonazepam: n (%)	5 (19.2%)
Vigabatrin: n (%)	5 (19.2%)
Cannabidiol: n (%)	4 (15.4%)
Phenytoin: n (%)	3 (11.5%)
Carbamazepine: n (%)	1 (3.8%)
Nitrazepam: n (%)	1 (3.8%)
Zonisamide: n (%)	1 (3.8%)
Ethosuximide: n (%)	1 (3.8%)
**Mean number of previous adjuvant therapies: range (mean ±** **standard deviation)**	0–3 (0.8 ± 1.0)
Neurosurgery for epilepsy: n (%)	3 (11.5%)
Ketogenic diet: n (%)	3 (11.5%)
Immunotherapy (corticosteroids or immunoglobulin): n (%)	3 (11.5%)
Vagus nerve stimulation: n (%)	5 (19.2%)
Adrenocorticotropic hormone: n (%)	2 (7.7%)

### Response to LCM treatment associated with other ASDs

**Figure 1:**
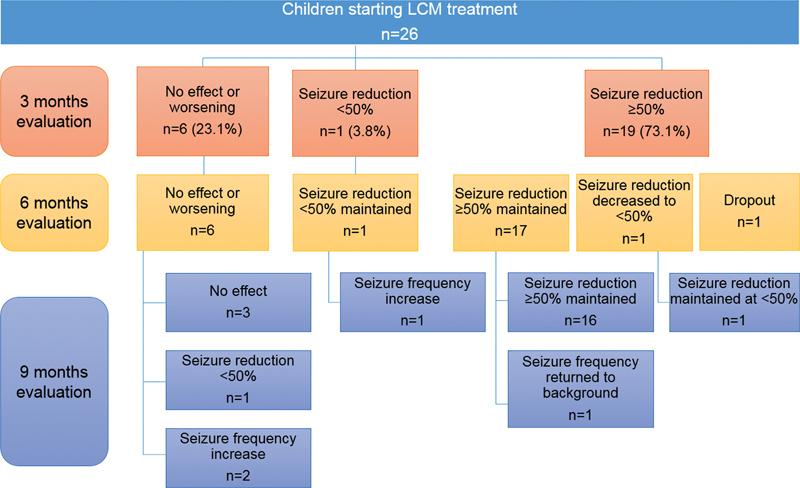
Clinical flowchart of the follow-up of children with refractory epilepsy receiving LCM after three, six, and nine months of treatment. Abbreviation: LCM, lacosamide.

The previous treatment modalities were not sufficient to control the seizures, and LCM was administered in the therapeutic scheme as an add-on. The mean initial dosage used was of 3.5 ± 1.5 mg/kg/day, divided into 1 to 3 daily administrations. The mean maintenance dosage was of 6.4 ± 2.3 mg/kg/day, and it was attained at 3 months of treatment. Lacosamide was used as a monotherapy in 2 patients. and as an add-on for the other 24 patients, who were under treatment with 1 to 4 (mean: 2.3 ± 1.6) other ASDs. Follow-up data were obtained for all 26 patients 3 months after the initiation of the LCM treatment, and for most of them ( *n* = 25), at months 6 and 9 as well ( Figure 1 ).

After 3 months of LCM administration, 73.1% ( *n* = 19) of the children had a reduction of > 50% in the frequency of seizures, and this clinical improvement was maintained in most of them ( *n* = 16) for the following 9 months. In total, 6 (23.1%) children did not respond to the LCM treatment or experienced an increase in seizure frequency, and 1 (3.8%) only showed a slight improvement. Among the 7 children who experienced no effect or worsening after 3 months of LCM administration, the same clinical profile was maintained for the following 6 months; 3 experienced an increase in the number of seizures, and 4 experienced no effect when followed-up at 9 months. And 2 children presented with mild adverse effects, such as behavioral changes ( *n* = 1) and somnolence ( *n* = 1).

To evaluate the association between ASDs and adverse effects, we classified the concomitant medications as sodium channel blockers (SCBs), which included phenytoin, oxcarbazepine, lamotrigine, and carbamazepine. We also determined 2 subgroups of patients according to their response to LCM: 17 children experienced clinical improvement after the LCM treatment and 9 children did not respond to the LCM treatment and/or had associated adverse effects. The demographic and clinical profiles of the subgroups that responded to LCM were compared. In the univariated analysis, we observed that among those who responded to LCM there was a higher prevalence of males, a lower number of concomitant ASDs, a lower percentage of patients using SCBs, and structural etiology associated or not to genetic alterations ( Table 3 ).

**Table 3:** Comparison of the demographic and clinical profiles of the children who responded to the lacosamide treatment after 3 months

Characteristics		Lacosamide responders ( *n* = 17)	Lacosamide non-responders ( *n* = 9)	*p*
Sex: n (%)	Male	11 (64.7%)	2 (22.2%)	0.097 ^a^
	Female	6 (35.3%)	7 (77.8)	
Epilepsy type: n (%)	Focal	16 (94.1%)	8 (88.9%)	1.000 ^a^
	Generalized	1 (5.9%)	1 (11.1%)	
Etiology: n (%)	Genetic	0 (0.0%)	2 (22.2%)	0.043 ^b^
	Genetic + structural	5 (29.4%)	2 (22.2%)	
	Structural	9 (52.9%)	1 (11.1%)	
	Unknown	3 (17.6%)	4 (44.4%)	
Age at seizure onset (years): mean ± standard deviation		3.8 ± 3.8	4.0 ± 4.4	1.000 ^c^
Age at the start of the lacosamide treatment (years): mean ± standard deviation		8.2 ± 3.9	9.7 ± 3.9	0.417 ^c^
Duration of epilepsy at the start of the lacosamide treatment (years): mean ± standard deviation		4.4 ± 3.0	5.7 ± 4.2	0.534 ^c^
Number of previous anti-seizure drugs: mean ± standard deviation		4.6 ± 2.4	4.4 ± 1.3	0.360 ^c^
Number of concomitant anti-seizure drugs: mean ± standard deviation		1.9 ± 1.1	3.0 ± 1.0	0.022 ^c^
Starting dose: mean ± standard deviation		3.5 ± 1.3	3.5 ± 1.8	0.645 ^c^
Maintenance dose: mean ± standard deviation		6.5 ± 2.2	6.2 ± 2.5	0.935 ^c^
Use of concomitant sodium channel blockers: n (%)		3 (17.6%)	7 (77.8%)	0.003 ^b^
Alteration on brain magnetic resonance imaging scan: n (%)		14 (87.5%)	3 (33.3%)	0.019 ^b^
Psychiatric comorbidities: n (%)		5 (55.6%)	2 (11.8%)	0.054 ^b^
Clinical comorbidities: n (%)		4 (44.4%)	8 (47.1%)	0.899 ^b^

Notes: ^a^ Fisher exact test; ^b^ Pearson Chi-squared test; ^c^ Mann-Whitney U test.

To evaluate the association between the concomitant use of SCBs and the response to LCM, a multiple logistic regression model was adjusted for possible confounders (sex). We observed that the concomitant use of SCBs decreased the probability of an effective response to the LCM treatment by ∼ 20 times ( Table 4 ). In addition, adverse effects were observed in 5 children, 3 of whom were making concomitant use of SCBs. There were 2 children with focal epilepsy who were not responsive to association of oxcarbazepine and LCM. However after oxcarbazepine suspention the response were improved and they was maintained with LCM monotherapy. They evolved with a good sustained response from the third to the ninth month of follow-up without adverse effects.

**Table 4:** Multiple logistic regression model evaluating the association between the concomitant use of sodium channel blockers and response to the lacosamide treatment

	Coefficient	Standard error of the coefficient	Wald test	*p*	Odds ratio
Sex (male)	2.290	1.279	3.205	0.073	9.876
Concomitant use of sodium channel blockers	-3.128	1.251	6.256	0.012	0.044
Constant	-1.164	1.703	0.467	0.494	0.312

## DISCUSSION

The efficacy and safety of LCM in adults with drug-resistant focal epilepsy have been established by randomized, multi-center, placebo-controlled studies. 12 13 14 Despite the use of LCM in adults, adolescents, and children older than 4 years of age, its efficacy and safety have not been established in children younger than that age. 15 16 17 The present study included children with refractory epilepsy who started LCM adjunctive therapy and responded very well to the treatment after 3 months (73.1%), with the majority of them showing a sustained response 9 months after LCM initiation, resulting in a final sustained response of 61.5% and corroborating the results of recent studies. 6 9 10 16 18 Other studies 16 19 have shown the long-term sustained efficacy of adjunctive LCM treatment; however, they did not report on the development of tolerability. The mean maintenance dose of LCM in our patients was of 6.4 ± 2.3 mg/kg/day, divided 1 to 3 times per day, as previously reported. 6 9 15 16 20

Our findings suggest that the response to LCM does not seem to depend on the number of previous ASDs, as previous treatment schemes included a similar number of ASDs for subgroups who responded and did not respond to LCM. Other authors 5 16 21 have shown an association between the LCM response and the number of previous ASDs; but, due to the small sample of the present study, it was difficult to validate this observation. In contrast, we observed fewer concomitant ASDs among those who responded to LCM, which is in line with the results of previous studies 8 16 21 22 that show an association between concomitant ASD and LCM efficacy.

Along with concomitant ASDs, it is important to discuss the action of SCBs. The association between SCBs and LCM efficacy remains controversial. A randomized, double-blinded, placebo-controlled trial by Farkas et al. 15 showed that the efficacy of LCM remained unaffected by SCB presence or absence. Other studies 7 8 16 23 have shown that the combination of LCM with non-SCB drugs would have a better synergistic action. We have shown a negative association between the concomitant use SCBs and LCM response, adjusted for confounders in a multiple regression analysis, in which children using SCBs had 20 times lower probability of responding to LCM ( *p* = 0.012; odds ratio [OR]: 0.044).

It is noteworthy that, in the present study, 2 patients with focal epilepsy did not respond to oxcarbazepine, but responded to the LCM monotherapy. This could be because, unlike traditional SCBs, LCM acts on slow sodium channels. 24 This rationale is in accordance with a study 25 evaluating a pre-specified historical cohort that showed the efficacy of LCM when other monotherapy agents were replaced with LCM in adults and adolescents (aged >16 years). Regarding etiology, the children with a structural lesion on MRI associated or not with genetic alterations ( *n* = 14), taking into account the EEG and the clinical semiology, were classified as having focal epilepsy and had better response to LCM. At first, the well-known good response to LCM in cases of focal epilepsy could explain this. 4 5 6 9 10 18 20 Nonetheless, not all patients with focal epilepsy had an abnormal MRI scan, and prognosis of good response to LCM cannot be based exclusively on that.

In addition, mild (somnolence, behavioral changes) or severe (worsening of the seizures) adverse effects were observed in 2 and 3 children respectively, and 3 of them (2 who presented with worsening of the seizures and 1, with somnolence) were under treatment with concomitant SCBs. Other studies 6 7 9 15 have reported that somnolence, vomiting, nasopharyngitis, dizziness, and pyrexia and other severe adverse effects were not related to the association between LCM and SCBs. Of note, most of them used other SCBs in combination with LCM; hence, the specific SCBs used in the present study, in combination with LCM, could increase the probability of developing adverse effects, similar to the finding by Halász et al. 13

The present study has some limitations. First, the number of patients included in our study was small, which eases the rigor of the conclusions. Moreover, due to that reduced number of patients, the possible associations of the LCM treatment with MRI or etiology were not performed. Second, due to the retrospective design of the study, data were obtained through a survey of medical records, and the raw numbers of daily seizures were not evaluated, as they was not available for most of the patients. Only the percentage of reduction in seizures was analyzed, as a categorical variable, once this piece of information was routinely recorded in the clinical sheets.

Despite the limitations, no other study has explored the effects of LCM in a pediatric population with refractory epilepsy in Brazil, which makes the present study quite valuable. Our findings support the use of LCM in pediatric patients as an add-on medication for refractory epilepsy, with few adverse effects, and an efficacy associated with fewer concomitant ASDs and absence of concomitant SCBs.
